# Cooperation in publicly funded reference material production

**DOI:** 10.1007/s00769-018-1349-1

**Published:** 2018

**Authors:** Håkan Emteborg, Doris Florian, Steven Choquette, Stephen L. R. Ellison, Maria Fernandes-Whaley, Lindsey Mackay, Pearse McCarron, Ulrich Panne, Sylvia G. Sander, Sook-Kyung Kim, Andrea Held, Thomas Linsinger, Stefanie Trapmann

**Affiliations:** 1 European Commission Joint Research Centre (JRC), Geel, Belgium; 2 National Institute of Standards and Technology (NIST), Gaithersburg, USA; 3 LGC, Teddington, UK; 4 National Metrology Institute of South Africa (NMISA), Pretoria, South Africa; 5 National Measurement Institute of Australia (NMIA), Lindfield, Australia; 6 National Research Council Canada (NRCC), Halifax, Canada; 7 Bundesanstalt für Materialforschung und –prüfung (BAM), Berlin, Germany; 8 International Atomic Energy Agency (IAEA) Environment Laboratories, Monaco City, Monaco; 9 Korea Institute of Standards and Science (KRISS), Daejeon, South Korea

## Introduction

The meeting was organised by the European Commission’s Joint Research Centre and held at the JRC-Geel site on 22–23 February 2018. It was a follow-up of a similar meeting held in 2009. The objective of the meeting was to exchange information about ongoing publicly funded reference material (RM) production, identify areas of interest for future specific RMs, including certified reference material (CRM) developments, investigate potential areas of collaboration, and to identify areas which may be of a lower importance in the future for a specific RM producer. The benefit of exchanging such information is to avoid duplication of efforts in RM production, make better use of public funds by potentially matching competencies, and to address problems that are common to publicly funded RM producers. Attendees included representatives of the following organisations: Bundesanstalt für Materialforschung und –prüfung (BAM), Germany; IAEA Environment Laboratories, Monaco; the Joint Research Centre (JRC), Belgium (formerly IRMM); the Korea Research Institute of Standards and Science (KRISS), South Korea; LGC, UK, the National Institute of Standards and Technology (NIST), USA; the National Measurement Institute of Australia (NMIA), Australia; the National Metrology Institute of South Africa (NMISA), South Africa; and the National Research Council Canada (NRCC), Canada. National Metrology Institute of Japan (NMIJ), Japan, and National Institute of Metrology (NIM), China, were invited but declined or were unable to attend. The remaining nine attendees participated physically or via videoconference. The full group of eleven RM producers corresponds to the major publicly funded RM producers according to the activity reports of the International Standards Organisation’s committee on reference materials (ISO-REMCO).

In preparation for the meeting, each participating organisation provided a list of current and planned projects. During the meeting itself, each institution presented its main fields of activity and offered additional information on its RM programme as required. Discussions not only addressed detailed technical questions but also dealt with questions on policy and legal status.

Additional meetings like this can help to assist in the overall prioritisation of RM production internationally. This meeting report discusses the drivers and approaches taken by publicly funded RM producers and analyses the areas of RM development currently being covered. A view of current areas of activity of the publicly funded RM producers is provided, and a summary of some future trends is given.

## Definitions

In the following text, the terms RM and CRM will be used extensively to describe the main product(s) of RM producers, together with relevant RM documentation, e.g. RM reports and certificates that accompany CRMs [1]. The terms RM and CRM are defined in ISO Guide 30:2015 as follows: “A RM is a material sufficiently homogeneous and stable with respect to one or more specified properties, which has been established to be fit for its intended use in a measurement process”; “A CRM is a RM characterized by a metrologically valid procedure for one or more specified properties, accompanied by a certificate that provides the value of the specified property, its associated uncertainty and a statement of metrological traceability” [2]. RM is the superordinate term, while CRM is a subordinate having a certified value and making it suitable for trueness checks. Metrological traceability is defined as a property of a measurement result whereby the result can be related to a reference through a documented unbroken chain of calibrations, each contributing to the measurement uncertainty [3]. For the remainder of this report, the publicly funded RM producers will simply be denoted “public RM producers”.

## Rationale for public funding of RM production

The public RM producers present at this meeting have a diverse history, some dating back to the middle of the nineteenth century and others formed in the late twentieth century. Legal status also varies widely, from wholly private company under government contract, through various government-owned company structures, to institutes which are formally part of the national or international civil service or government structure. These different structures place differing constraints on each organisation. In general, the publicly funded RM producers have been asked by their relevant authorities to develop and supply analytical laboratories with RMs (usually certified reference materials—CRMs) in gaseous, solid, or liquid form, thereby providing quality assurance tools for different measurement purposes. Depending on the country and the legislative context, this authorisation is more or less formalised. Some are cited in national legislation or in national or international standards for implementation or validation of specific measurement processes. In this way, the public sector enables provision of crucial RMs by funding their public RM producers to support industry or to ensure consumer safety through legislation. Citation in legislation provides a general basis for public funding and sometimes confers a specific responsibility. For example, National Metrology Institutes have specific responsibilities to provide and maintain national measurement standards, in order to underpin legislation requiring metrological traceability to national measurement standards. In other cases, the institute is named in government policy statements, rather than a specific statute.

However, legislation and policy do not usually specify the particular RMs and CRMs to be produced by an entity, that decision is typically driven by wider considerations. One key rationale for discretionary public funding, common to public RM producers present, is the identification of an established need that cannot be fulfilled economically by commercial activity despite a substantial benefit to society. For example, the cost of production at the required quality or the capital cost of specialised facilities may not be recoverable from realistic sales income. This is often true of certified *matrix* reference materials, (which frequently incur high production costs) and whose annual sales volume may be low as they see most use in occasional, but crucial, validation studies. It can also be true of apparently simpler materials when production cost is high and use is moderate. An example is the production of calibration materials for sports doping, which must be produced to the highest international standards to ensure international acceptance, yet whose development and production cost cannot be met in full by the limited number of agencies performing monitoring in this area. In yet other cases, there may be a unique technical requirement; for example, metrological traceability of property values in commercially available calibrants may require calibration against CRMs produced by public RM producers, where efforts have been invested in establishing traceability to the SI. In other instances, public agencies may be better able to handle special licensing or control requirements; for example pure calibrants of illicit drugs and/or doping substances. For the reference material producers present at the meeting, however, a common consideration is that publicly funded RM production is not generally undertaken where it would compete with technically sound, commercially viable activity by commercial producers. Accreditation to ISO 17034, with metrological traceability to relevant standards where appropriate, can help to demonstrate technical validity in commercial production [1].

The public and commercial RM producers complement each other in providing crucial tools to measurement laboratories that need to fulfil a very wide variety of measurement tasks as accurately as possible within the boundaries of fitness for purpose.

## Ongoing projects, future trends and challenges, new certified reference material developments

A key reason for holding a public RM producers’ meeting in the first place is to identify overlapping interests in order to make better use of limited public funds and technical competence related to the human resources. It is usually better that two different types of CRM exist than that two closely similar CRMs are produced, unless there is sound justification for duplication. Exchanging information on legislative drivers can help to reduce unnecessary duplication. For example, many of the analytes related to food safety have broadly similar legislative limits in different jurisdictions. By exchanging information on legislative requirements, publicly funded producers can ensure that an RM produced by one producer is suitable for use in a broad range of jurisdictions, reducing the call on public funds worldwide. Conversely, a laboratory located anywhere in the world and carrying out measurements for the implementation of specific legislation can use any reference material as long as it is certified for the (legally) relevant property in a similar matrix at suitable concentration. Consequently, since development of high-quality CRMs requires investment of money, time, and human resources; duplication should be avoided.

Tables 1, 2, 3, 4 and 5 provide schematic overviews of the current activities of the publicly funded RM producers represented at the meeting. The tables specify the matrices per row and the properties intended to be certified per column. This compilation only shows new materials under production at the different centres and is not an exhaustive list of all of the CRMs available from these RM producers. The letter in each box represents the different public RM producers as explained in the footnote of each table. Generally, only one or two producers are targeting similar matrix and to-be-certified property combinations, with some exceptions. This effectively shows that the public RM producers already complement each other in many respects. In some cases, identification of two producers refers to projects that are performed in collaboration, e.g. NIST and the JRC are collaborating on producing an RM suspension of nanomaterials to be certified for their zeta potential. In other fields, certain overlaps exist where it can be a question of replacement of legacy materials (i.e. trace elements in food) or areas where multiple target parameters exist in combination with many different commodities (e.g. mycotoxins). This particular area can also be of interest for targeted production of RMs where certain commodities are not consumed (or analysed) on a global level (although the area in general is already covered). However, for both of these areas, apparent overlaps are generated by the broad categories (“elements in food”), in some cases simply due to shipping and biosecurity problems (e.g. for aflatoxins).

Most public RM producers are focussing on specific categories of materials, e.g. algal biotoxins, genetically modified organism (GMO) materials, certified gas mixtures, steels and alloys, environmental CRMs or CRMs for nutritional supplements. In that respect, many of the producers will continue to refine and develop new materials that are typical for their areas of competence.

The current portfolio of RMs from the public RM producers present at this meeting can be found on their respective websites [4–12].

For all producers, new challenges are arising in areas of (C)RMs for species identification based on DNA sequencing, nanoparticles embedded in food or cosmetics, micro-plastics in food and environment, reference data for comparing algorithms and bioinformatics data, materials for fine atmospheric dust, RMs for allergens and clinical reference materials related to an ageing population. New requirements also pose challenges; most of the publicly funded producers present see challenges in addressing issues of commutability for clinical reference materials.

## Follow-up

It was agreed among the public RM producers at this meeting that a yearly follow-up will be organised through videoconferencing given the large geographical spread of participants. About every third year, a face-to-face meeting should be organised. Exchange of scientists was another possibility that was discussed. Additionally, the producers agreed to communicate between themselves on an ongoing basis regarding RMs that they are not intending to replace. The latter aspect is important for two reasons; firstly abandoning replacement of legacy materials liberates resources for new developments to resolve a complex problem that requires substantial investment in R&D at the outset. Secondly, the discontinuation of a specific RM may cause problems for particular measurement communities. In that respect, it is also of value for RM producers to inform laboratories in advance of RM depletion that similar CRMs from another RM producer may cover the need. The COMAR database is an online tool to search for suitable CRMs/RMs covering a large number of producers [13].

## Figures and Tables

**Table 1:** Ongoing RM development activities in the area of food and feed reference materials, matrices are given per row and target properties per column

	Vitamins	Elements	Proximates	Fatty acids	Amino acids	DNA sequence	DNA	Cell count	Pesticides	PAHs	Nitrate	Drugs	Radionuclides	Mycotoxins	Algal toxins	Species identification	GMO	Capsaicin	SO_2_	Ethanol content

Solution						J	J					C		S, C	C					
Plant	N, K	N, B, S, K	N	N					J	N, K	L	C	K, U	N, S, C, K, J			J	K		
Animal tissues	K	L, J	L	N				J				S, J, C	K, U		C, U					
Water		L													C					
Drink																			L	L
Processed food feed		L, J	L										U							
Infant formula	K	K		K	S															
Tablet	N															N				

A = NMIA; B = BAM; C = NRC; J = JRC; K = KRISS; L = LGC; N = NIST; S = NMISA; U = IAEA

*PAH* polycyclic aromatic hydrocarbons, *GMO* genetically modified organism materials

**Table 2:** Ongoing RM development activities in the area of health-related (clinical) reference materials, matrices are given per row and target properties per column

	DNA copy cone	DNA mass cone	DNA sequence	Proteomics	Total cell count	Proteins	Labelled proteins	Enzymes	Glycans	Hormones	Fatty acids	Elements	Vitamins	Drugs	Bacterial toxins	Ethanol

Solution	N, A	N	N	N	N	N	N		N						J	L, A
Pure compounds						C		J		C, A				L, A		
Urine						N				A				A		
Serum/plasma		K				N				N	N	L	N, K			
Blood												L		L		
Hemolysate						K										
Other human fluids						J										

A = NMIA; B = BAM; C = NRC; J = JRC; K = KRISS; L = LGC; N = NIST; S = NMISA; U = IAEA

**Table 3:** Ongoing RM development activities in the area of environmental reference materials, matrices are given per row and target properties per column

	Elements	Chemical species	Stable isotopes	Radionuclides	PAH	PCB	BFRs/HBCDD	PFAA	PFR	Pesticides	OC	MOH	Other organics	Hazardous air pollutants	HC/greenhouse gases	Sulfur compounds	Hydrochloric acid Hydrofluoric acid

Solution		N, L	N, L														
Dust (PM_10_ PM_2,5_)	J						N	N	N								
Soil, sediment rocks	N, U		U	K, U	B, N, U	N, U	N, J, U	N, A		N	U	B, U					
Crude oil													N				
Animal tissue	N, U, J			U	U	U	J, U				J, U	U					
Gas			U											S, K	S, N, U	S, K	K
Water	K, J		U	U													
Organic matter			U	U													

A = NMIA; B = BAM; C = NRC; J = JRC; K = KRISS; L = LGC; N = NIST; S = NMISA; U = IAEA

*PAH* polycyclic aromatic hydrocarbons, *PCB* polychlorinated bisphenyls, *BFR* brominated flame retardants, *HCBDD* hexabromocyclododecane, *PFAA* perfluoroalkyl acids, *PFR* phosphorus flame retardants, *OC* organic carbon, *MOH* mineral oil hydrocarbons, *HC* Hydrocarbons

**Table 4:** Ongoing RM development activities in the area of industrial reference materials, matrices are given per row and target properties per column

	Phthalates	Leachability	PAH	FAMEs	Elements	SY124 EU-fiscal marker

Wood					N	
Glass		B			N, B	
Oil					N	
Plastics	N		B, K			
Metals/alloys					N, B, J	
Ceramics					B	
Gas oil						J

A = NMIA; B = BAM; C = NRC; J = JRC; K = KRISS; L = LGC; N = NIST; S = NMISA; U = IAEA

*PAH* polycyclic aromatic hydrocarbons, *FAMEs* fatty acid methyl esters

**Table 5:** Ongoing RM development activities in the area of physical property reference materials, matrices are given per row and target properties per column

	Particle size	Layer thickness	Transmittance	Fusion enthalpy	Quantum yield	Enthalpy of fusion	Particle shape	Zeta potential

Powder	C						C	C
Suspension	N, B, J				B		J	J/N
Tissue		N						
Metal on quartz			N			L		
Si/Ge				N				
Solution			N					
Film			B					

A = NMIA; B = BAM; C = NRC; J = JRC; K = KRISS; L = LGC; N = NIST; S = NMISA; U = IAEA

## References

[1] ISO 17034 (2016) General requirements for the competence of reference material producers. ISO, Geneva

[2] ISO Guide 30 (2015) Reference materials—selected terms and definitions. ISO, Geneva

[3] ISO/IEC Guide 99 (2007) International vocabulary of metrology—basic and general concepts and associated terms (VIM). ISO, Geneva 10.1016/j.clinbiochem.2008.09.007 19863914

[4] http://www.kriss.re.kr/eng/rnd/rnd03_2.html.

[5] https://www.nist.gov/srm. Accessed 20 July 2018

[6] www.nrc.ca/crm. Accessed 20 July 2018

[7] http://www.nmisa.org. Accessed 20 July 2018

[8] https://crm.jrc.ec.europa.eu/. Accessed 20 July 2018

[9] https://www.lgcgroup.com/our-science/national-measurement-laboratory/#.WstY-v5lJ9A. Accessed 20 July 2018

[10] https://rrr.bam.de/RRR/Navigation/EN/Reference-Materials/uebersicht-reference-materials.html. Accessed 20 July 2018

[11] https://nucleus.iaea.org/rpst/referenceproducts/ReferenceMaterials/index.htm. Accessed 20 July 2018

[12] http://www.measurement.gov.au/Services/CertifiedReferenceMaterials/Pages/default.aspx. Accessed 20 July 2018

[13] https://www.comar.bam.de/home/login.php. Accessed 20 July 2018

